# SARS-CoV-2-Specific T Cell Immunity in HIV-Associated Kaposi Sarcoma Patients in Zambia

**DOI:** 10.1155/2022/2114285

**Published:** 2022-07-28

**Authors:** Owen Ngalamika, Marie Claire Mukasine, Patrick Kamanzi, Musonda Kawimbe, Aaron Mujajati, For Yue Tso, Salum J. Lidenge, Chibamba Mumba

**Affiliations:** ^1^Dermatology and Venereology Division, Adult University Teaching Hospital, University of Zambia School of Medicine, Lusaka, Zambia; ^2^HHV-8 Molecular Virology Laboratory, University Teaching Hospital, Lusaka, Zambia; ^3^Carepeak Specialist Clinic, Lusaka, Zambia; ^4^Department of Interdisciplinary Oncology, Louisiana State University Health Sciences Center, New Orleans, LA, USA; ^5^Ocean Road Cancer Institute, Dar-es-Salam, Tanzania; ^6^Muhimbili University of Health and Allied Sciences, Dar-es-Salam, Tanzania; ^7^Department of Pathology and Microbiology, University of Zambia School of Medicine, Lusaka, Zambia

## Abstract

Severe acute respiratory syndrome coronavirus type 2 (SARS-CoV-2) virus is the cause of coronavirus disease 2019 (COVID-19). It has caused millions of infections and deaths globally over a 2-year period. Some populations including those living with HIV and/or cancer are reported to be at a higher risk of infection and severe disease. HIV infection leads to a depletion of CD4^+^ T cells which impairs cell-mediated immunity and increases the risk of malignancies such as Kaposi sarcoma (KS) and viral infections such as SARS-CoV-2. However, several other factors including level of immunosuppression and chemotherapy may also affect the immune response against SARS-CoV-2. In this study, we investigated factors affecting SARS-CoV-2-specific T cell immunity towards the spike, nucleoprotein, membrane protein, and other open reading frame proteins in individuals with HIV-associated KS. The KS patients were SARS-CoV-2 seropositive with detectable T cell responses, but had no history of symptomatic SARS-CoV-2 infection. We observed that the T cell responses increase from baseline levels during follow-up, with responses towards the NMO peptide pool being statistically significant. Low CD4 counts below 200 cells/*μ*l were associated with lower SARS-CoV-2-specific T cell responses. Cancer chemotherapy and KS T staging did not have a significant effect on the T cell responses.

## 1. Introduction

The recently discovered SARS-CoV-2 virus is the cause of the ongoing coronavirus disease 2019 (COVID-19) pandemic [1]. There have been hundreds of millions of infections due to SARS-CoV-2 and millions of deaths due to COVID19 since the first few cases were reported in late 2019 in China [2]. SARS-CoV-2-infected individuals with HIV/AIDS-associated malignancies remain at a higher risk of morbidity and mortality due to the immunosuppression associated with both the HIV infection and the malignancy [3, 4].

Despite HIV-infected individuals being at a higher risk of infection with SARS-CoV-2, a high proportion is reported to be asymptomatic when they get infected [5]. This reduces the likelihood of detecting SARS-CoV-2 infections among the HIV-infected and isolating these individuals from the general population. Also, due to the immunosuppression associated with HIV disease, these individuals are less likely to clear the virus, and this may lead to prolonged infection and a higher probability of mutation of the virus in these hosts [6]. More specifically, the HIV virus infects CD4^+^ T cells and causes a depletion of these cells with a resultant deficiency in cell-mediated (T cell) immune responses [7].

Both HIV and Kaposi sarcoma (KS) are associated with a defective cell-mediated immune response [8, 9]. KS is an AIDS-defining angio-proliferative malignancy commonly affecting the skin, lungs, and gastrointestinal tract and is caused by the Kaposi sarcoma-associated herpes virus (KSHV) [10]. A defective cell-mediated immune response, largely attributed to HIV infection, is insufficient in controlling KSHV replication, hence increasing the risk of KS development [10]. KS is the most prevalent HIV-associated malignancy and is among the top 10 cancers in most sub-Saharan Africa countries (GLOBOCAN, 2022). Despite previous studies observing no increased risk of COVID19 among patients presenting with some of the most common cancers [11], individuals with HIV-associated KS may be an exception due to the role of HIV in KS pathogenesis and in compromising T cell immunity in this population.

T cell immunity is crucial in the body's defense against intracellular organisms such as viruses and the body's fight against cancer [12]. Key players in T cell immunity include CD4 and CD8 (cytotoxic) T cells. These cells recognize human leukocyte antigen-peptide complexes and kill cancerous cells and cells infected by viruses [13]. A deficiency in this immunity may predispose an individual to chronic viral infections and cancer. Studying SARS-CoV-2-specific cell-mediated immunity in potentially vulnerable populations such as HIV-associated KS patients may be important in the development of strategies to control infections and also prevent emerging variants. Also, other factors including cytotoxic cancer chemotherapy administered to KS patients may potentially worsen the immunosuppression which may further dampen the anti-SARS-CoV-2 T cell response.

This study was aimed at longitudinally studying SARS-CoV-2-specific T cell immunity in HIV-associated KS patients. We investigated how T cells in these individuals are likely to recognize and clear SARS-CoV-2 infection, and also how factors including disease stage, level of immunosuppression, and treatment with cancer chemotherapy are likely to affect this T cell immunity. Our study is unique because it was done in an African setting where HIV-associated malignancies are common, and where SARS-CoV-2-specific T cell immunity has not been well-characterized in cancer patients.

## 2. Materials and Methods

### 2.1. Study Design and Participants

This was a prospective cohort study to investigate SARS-CoV-2-specific T cell immunity and factors affecting this immunity in SARS-CoV-2-seropositive individuals with HIV-associated KS. The participants were recruited from the University Teaching Hospital (UTH) in Lusaka, Zambia. The UTH is the largest referral public hospital in the country and receives patients from other hospitals in the capital city and other parts of the country. After obtaining informed consent, clinical and sociodemographic information was collected followed by collection of venous whole blood. All the participants were asymptomatic but seropositive for SARS-CoV-2 at the time of recruitment. Past infection with SARS-CoV-2 was determined at the time of recruitment using a rapid antibody test (PANBIO COVID-19 IgG/IgM rapid test). The study participants were followed up for approximately 8 weeks after recruitment. During the follow-up period, the participants also underwent cytotoxic cancer chemotherapy for KS. Ethical approval was obtained from the University of Zambia Biomedical Research Ethics Committee (REF. No. 019-017-18) and from the National Health Research Authority (Ref No: NHRA00001/17/09/2021).

### 2.2. Sampling

16 ml of venous whole blood was collected from all the participants at both baseline and at follow-up. A portion of the whole blood was used for CD4 counting and another portion for T cell immunophenotyping by flow cytometry, and then, the rest underwent centrifugation to separate plasma. A portion of the plasma was used for HIV viral load quantification, while the rest was stored at -80°C. After separating plasma, the remaining cellular component of the blood was then subjected to density gradient centrifugation to separate peripheral blood mononuclear cells (PBMC) for subsequent interferon gamma enzyme-linked immunospot (IFN*γ*-ELISpot) assays.

### 2.3. CD4 Counting and HIV Viral Loads

The BD TriTest kit (BD biosciences) was used to quantify CD4 counts on a 4-color BD FACSCalibur (BD Biosciences). The Aptima HIV-1 Quant Dx assay kit was used to determine HIV viral loads on a Hologic Panther (Hologic) according to the manufacturer's protocol.

### 2.4. ELISpot Peptides

PBMCs were stimulated with the peptide pool covering the SARS-CoV-2 S1 subunit of the spike (S) protein which contains the receptor binding domain (RBD) and is important for viral entry into cells [14], the SARS-CoV-2 nucleoprotein (N) which is important for viral replication and packaging [15, 16], the membrane (M) protein which is important in production of virus-like-particles [17], and other open reading frame (O) proteins which were obtained from MABTECH AB (Sweden). The NMO was a combined peptide pool.

### 2.5. ELISpot Assay

An IFN*γ*-ELISpot assay, which detects IFN*γ*-secreting cells at a single cell level, was conducted. Both at baseline and after 8 weeks of follow-up, 96-well precoated plates (MABTECH AB, Sweden) were used to conduct the assays in duplicate. A total number of 250,000 freshly isolated live PBMCs were added to each well and stimulated with peptides. Responses to 2 *μ*g/ml of the peptide pools were measured. Anti-CD3 monoclonal antibody was the stimulant in the positive control well, while dimethyl sulfoxide (DMSO) was used instead of peptides in the negative control wells. Co-stimulation of the T cells was done by adding anti-CD28 in all the wells. The plate was incubated at 37°C in a humidified incubator with 5% CO2 for 24 hours. Detection antibody was added followed by the development of spots the next day (on day 2), according to the manufacturer's protocol. Counting of spots was done using an AID ELISpot reader (Autoimmun Diagnostika) (Figure S1). An average of the spots in the 2 wells for each peptide pool was calculated and used for the final analysis. A final count of at least 55 spot-forming units (SFU) and 4 times the count in the negative control for each participant were considered to be a positive response.

### 2.6. Flow Cytometry

We used CD3-APC-Vio770, CD4-PE, CD8-PE-Vio770, and CD45RO from Miltenyi Biotec (Germany) and CD197-CCR7-FITC from BD Biosciences (Belgium) to stain the whole blood for 30 minutes. A 6-color BD FacsVerse instrument (BD Biosciences) was used to conduct the flow cytometry, and Flow Jo (version 10) was used to analyze the data. The gating strategies are shown in Figure S2.

### 2.7. Statistical Analysis

Statistical analysis was done using STATA version 17. We used summary statistics to analyze the baseline characteristics. Continuous variables are presented as median and interquartile range, while categorical or binary variables as percentages. The Wilcoxon rank-sum test was used to compare the counts or other continuous variables between two groups. The Wilcoxon matched-pairs signed-rank test was used to compare the counts between baseline and follow-up time points. The Spearman's rank correlation was used to determine whether there was any significant correlation between continuous variables. A *p* value of < 0.05 was considered statistically significant.

## 3. Results

### 3.1. Characteristics of Study Participants

We recruited and prospectively followed up 22 SARS-CoV-2 seroreactive, mainly SARS-CoV-2 unvaccinated, individuals with newly diagnosed HIV-associated KS. The study participants were treatment (cytotoxic cancer chemotherapy)-naïve at the time of recruitment, and the majority (about 91%) was on antiretroviral therapy (ART). None of the study participants had previously been hospitalized due to COVID19, and only 4.5% had experienced COVID19 symptoms. The rest of the baseline characteristics are shown in Table 1. After 8 weeks of follow-up, the participants had received a median of 4 doses [IQR: 3-6] of cytotoxic cancer chemotherapy as part of standard care for KS. The chemotherapy is comprised of 1 to 3 drugs/doses (doxorubicin, bleomycin, and vincristine) administered every 3 weeks. Three (3) KS patients died during the follow-up period due to advanced KS disease, and another one was admitted with severe visceral KS disease at the time of follow-up and hence could not be sampled. Four (4) other participants were lost to follow-up. We therefore successfully followed up 14 study participants out of the recruited 22.

### 3.2. SARS-CoV-2-Specific T Cell Responses at Baseline Compared to Follow-Up

**Figure 1:**
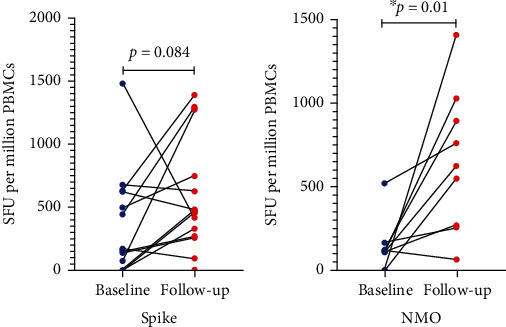
SARS-CoV-2-specific T cell responses between baseline and follow-up. (a) T cell responses against spike S1 subunit at baseline and follow-up. (b) T cell responses against NMO at baseline and follow-up.

At baseline, 18/22 (81.8%) of the participants had detectable T cell responses towards S1 subunit of spike, while 13/16 (81.3%) of the participants had detectable T cell responses towards NMO. At follow-up, 13/14 (92.9%) of the followed-up participants had detectable T cell responses against spike S1 subunit, while 14/14 (100%) of followed-up individuals had detectable T cell responses against NMO. There was an increase in T cell responses against both spike and NMO peptide pools after 8 weeks of follow-up, with a statistically significant increase for the NMO peptide pool at the time of follow-up (*p* = 0.01) (Figure 1).

### 3.3. Effect of CD4 Counts on SARS-CoV-2-Specific T Cell Responses

**Figure 2:**
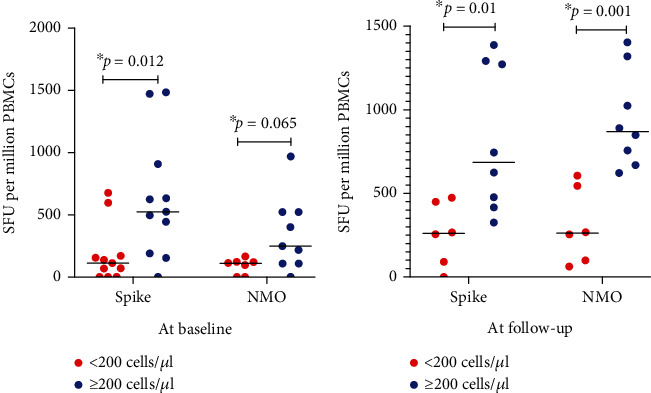
SARS-CoV-2-specific T cell responses by CD4 counts. (a) SARS-CoV-2-specific T cell responses against Spike S1 subunit and NMO peptide pools by CD4 count categories at baseline. (b) SARS-CoV-2-specific T cell responses against Spike S1 subunit and NMO peptide pools by CD4 count categories at follow-up.

To investigate whether the level of immune suppression in newly diagnosed HIV-associated KS patients affected the SARS-CoV-2 T cell responses, we categorized the study population into those with CD4 count above or below 200 cells/*μ*l and compared the responses between these two subgroups. Overall, there was a statistically significant increase in CD4 counts from baseline levels after 8 weeks of follow-up (199 cells/*μ*l [103-339] *vs*. 206 cells/*μ*l [147-465]; *p* = 0.025). At baseline, we observed statistically significant higher T cell responses towards the spike S1 subunit peptide pool in individuals with higher CD4 counts compared to those with low CD4 counts as expected (*p* = 0.012) (Figure 2). There was a trend towards an increase in T cell responses against the NMO pool in individuals with higher CD4 counts compared to those with low CD4 counts; however, this difference did not reach statistical significance (*p* = 0.065). During follow-up, responses towards both spike S1 subunit peptide pool (*p* = 0.01) and NMO peptide pools (*p* = 0.001) were statistically significantly higher in individuals with higher CD4 counts than those with low CD4 counts. Also, there was a statistically significant positive correlation between CD4 counts and T cell responses towards spike S1 subunit and NMO peptide pools at baseline (*r* = 0.61, *p* = 0.01 and *r* = 0.61, *p* = 0.012, respectively) and follow-up (*r* = 0.65, *p* = 0.011 and *r* = 0.68, *p* = 0.01, respectively) (Figure S3).

### 3.4. Effect of KS Stage, Chemotherapy, and Gender on SARS-CoV-2-Specific T Cell Responses

The KS patients were categorized according to the AIDS Clinical Trial Group T staging criteria which is based on extent of the tumor. T0 stage often represents less extensive disease and less severe immunosuppression, while T1 stage often represents more advanced disease and more severe immunosuppression. About 59% of the participants had the advanced T1 stage at baseline. We observed no significant differences in T cell responses between those with the T0 stage and those with the T1 KS stage at baseline (Table S1). Also, there was no significant difference in T cell responses between males and females. Since we had a narrow age range, we did not investigate the effect of age on the T cell responses.

During the follow-up period, the participants received cytotoxic cancer chemotherapy for KS. At our facility, KS patients often receive 2-3 chemotherapy drugs at each cycle for 6 cycles and a total of about 12-18 doses. Given the two-month follow-up period, the participants were still undergoing chemotherapy at the time of follow-up. The number of doses received ranged from 3 to 8 depending on clinical eligibility and consistency in patient's presentation to the hospital for drug administration. There was no significant correlation between the number of doses administered during the follow-up period and the responses to spike and NMO peptide pools (Table S2).

## 4. Discussion

This study aimed at investigating SARS-CoV-2-specific T cell responses, and factors affecting these immune responses, in individuals with newly diagnosed HIV-associated KS because it is the most common HIV-associated malignancy and one of the most prevalent malignancies in our setting. It was hypothesized that T cell responses may be weaker in individuals with lower CD4 T cell counts than those with higher CD4 counts [18]. Also, cytotoxic cancer chemotherapy administered to KS patients was expected to dampen the T cell responses as it often causes an immunosuppression [19].

We observed that, at baseline, over 80% of the study participants had detectable SARS-CoV-2-specific T cell responses towards the two peptide pools. After 8 weeks of follow-up, T cell responses towards both spike and NMO peptide pools increased, with a statistically significant increase towards the NMO peptide pool. The observed improvement in T cell responses after 8 weeks of follow-up could be attributed to reconstituted immune system as a result of HIV control by ART and/or T cell responses that develop slowly over time due to a dysregulated and senescent immune system as has been observed in the elderly who also have a senescent immune system [20, 21]. Since the majority of our study participants had no history of experiencing symptomatic SARS-CoV-2 infection or undergoing a PCR diagnosis, it is not known when they actually got infected and how the immune response changed over time from the time of infection. However, they were recruited after a third COVID19 wave in Zambia, which was dominated by the delta variant, and was approximately 8-12 weeks prior to commencing this study. Therefore, it is possible that the observed increase in the T cell response may be due to a slow response rather than sustained improvement over time. If that is the case, this slow response and asymptomatic nature of the SARS-CoV-2 infection warrants further investigation. Individuals with HIV-associated KS are likely to harbor the virus for a longer period and may thus be a source of new variants as the virus has enough time to mutate [22]. Also, most of the HIV-associated KS patients may be asymptomatic and thus may be a source of asymptomatic viral transmission in the general population.

The level of immunosuppression appears to be a major factor contributing to weaker T cell responses in individuals with HIV-associated KS. We observed that individuals with CD4^+^ T cell counts below 200 cells/*μ*l had weaker SARS-CoV-2-specific T cell immunity. This observation is in agreement with previous studies that have reported that lower CD4^+^ T cell counts are associated with suboptimal SARS-CoV-2 T cell and humoral responses in people living with HIV [23, 24]. CD4^+^ T cells are an important component of adaptive immunity in the control of viral infections such as SARS-CoV-2. These cells can have a direct cytotoxic effect on infected cells, can help B cells mature and produce neutralizing antibodies, and can also help support the differentiation and function of CD8^+^ cytotoxic T cells [25, 26]. Hence, a deficiency or impaired function of these cells can have a detrimental effect in the control of viral infections such as SARS-CoV-2. This underscores the need to commence ART early in individuals infected with HIV, including those presenting with HIV-associated KS, in order to prevent HIV-induced depletion of CD4^+^ T cells which may lead to weaker control of SARS-CoV-2 infection.

Individuals with HIV-associated KS present at different stages of disease, according to the AIDS Clinical Trials Group (ACTG) staging criteria. Also, these individuals undergo treatment with cytotoxic cancer chemotherapy to control the KS tumors. We observed no association between the T stages (T0 *vs*. T1) reflecting the extent of KS disease, with T cell responses at baseline before commencing chemotherapy in our study cohort. This was similar to a previous study on cancer patients by Fendler et al., where it was observed that cancer staging was not correlated with SARS-CoV-2 immune responses [27]. Similarly, the number of chemotherapy doses administered to KS patients did not associate with T cell responses measured. Overall, compared to baseline responses, the T cell responses improved during follow-up period despite chemotherapy in the entire cohort. This was also similar to a study where both humoral and T cell responses were evaluated in vaccinated patients with lymphoid malignancies after receiving anti-CD20 and chemotherapy treatment, and it was observed that the majority had detectable T cell responses (>80%), while the humoral responses were detected in fewer individuals (<15%) [28]. It therefore appears from our study and others that SARS-CoV-2 specific T cell immunity is not significantly affected by cancer stage and chemotherapy. This is an important observation because cytotoxic cancer chemotherapy is a key component in the management of advanced HIV-associated KS patients, especially in resource-limited settings. However, this observation may be due to the study design as we did not compare individuals who received chemotherapy to those who did not, as all the participants were eligible for chemotherapy. Nevertheless, the participants were on ART, which led to an improvement in CD4 counts during treatment, and can partly explain the improvement in the T cell response during chemotherapy. Also, the participants were followed up and sampled at least 3 weeks after receiving chemotherapy, and hence, their T cells may have recovered from the effects of chemotherapy at the time of sampling. On the other hand, there was no significant difference in T cell responses between males and females in our study cohort, although a larger sample size may be required as the majority of the KS patients were males, as KS affects more males than females.

The presence of cross-reactive humoral and adaptive immune responses against SARS-CoV-2 has been previously reported in our settings [29, 30]. The lack of COVID-19 symptoms and hospitalization for the majority of this cohort could be due to cross-protective immune response against SARS-CoV-2 infection. Alternatively, immunosuppression associated with HIV-associated KS could be the reason for the asymptomatic nature of SARS-CoV-2 infection in the cohort. This is supported by the fact that COVID19 disease symptoms are a manifestation of a heightened immune response, as observed by a previous study that reported that asymptomatic individuals had a weaker immune response to SARS-CoV-2 infection, lower levels of pro- and anti-inflammatory cytokines, and prolonged viral shedding [31].

## 5. Study Limitations

Because our cohort of HIV-associated KS patients was serologically diagnosed, we could not estimate the time of SARS-CoV-2 infection. This is important because immune responses, particularly T cell responses, are known to decrease with time, and so the lack of association in some comparisons could be due to loss of response as a function of time and not the lack of association. However, we observed an increase in T cell immunity during the follow-up period, which could indicate more recent infection at the time of recruitment. The asymptomatic infections in HIV-associated KS patients also affected the study design in that we could not compare them to HIV-infected individuals without KS who were usually symptomatic or diagnosed by PCR. Also, the small sample size in our study might have limited the power to detect a difference for some of the variables. In addition, we did not follow-up both seropositive and seronegative individuals to investigative any potential changes in serostatus. Nevertheless, this study has highlighted the presence of SARS-CoV-2 specific T cell responses and how these responses change over time in HIV-associated KS patients.

## 6. Conclusion

We report for the first time the presence of SARS-CoV-2 specific T cell responses in individuals with newly diagnosed HIV-associated KS in sub-Saharan Africa. The majority of these individuals have asymptomatic infection. Low CD4 counts below 200 cells/*μ*l are associated with much lower SARS-CoV-2-specific T cell responses. Cytotoxic cancer chemotherapy and extent of KS disease do not have a significant effect on T cell responses against SARS-CoV-2.

## Figures and Tables

**Table 1:** Baseline characteristics of study participants.

Median age in years	37.5 [32-42]
Males	17 (77.3%)
BMI in kg/m^2^	24.3 [21.1-26.1]
CD4 count in cells/*μ*l	199 [103-339]
HIV viral load in copies/ml	0 [0-42.5]
On ART	20 (90.9%)
Symptomatic for COVID19	1 (4.5%)
Vaccinated at baseline	2 (9.1%)
KS ACTG T1 stage	13 (59.1%)

BMI: body mass index in kg/m^2^; ART: antiretroviral therapy; KS: Kaposi sarcoma; ACTG: AIDS Clinical Trials Group.

## Data Availability

The data that support the findings of this study are available from the corresponding author [O.N.], upon reasonable request.

## Supplementary Materials

Supplementary Materials Figure S1. T cell responses in individual wells showing the spot-forming units. (a) Responses in a well for one of the peptide pools under investigation; (b) a negative control well where DMSO was added instead of peptide pools; (c) a well showing responses in the positive control well where stimulation of CD3, a T cell co-receptor, was done. Figure S2. Gating strategies used for immunophenotyping by flow cytometry. (a) Forward and side scatter with a gate on lymphocytes; (b) gating on CD3^+^ cells; (c) gating of CD4^+^ and CD8^+^ cells among the CD3+ cells; (d) subsets among the CD4 or CD8 cells including effector memory (Q1), central memory (Q2), naïve (Q3), and effector (Q4) cells. At least 100,000 events were collected in the lymphocyte gate. Figure S3. Correlation between CD4 counts with SARS-CoV-2-specific T cell responses. (a) a significant positive correlation between CD4 counts and T cell responses to spike at baseline; (b) a significant positive correlation between CD4 counts and T cell responses to NMO at baseline; (c) a significant positive correlation between CD4 counts and T cell responses to spike at follow-up; (d) a significant positive correlation between CD4 counts and T cell responses to NMO at follow-up. Table S2. Correlation between chemotherapy doses received and responses to SARS-CoV-2 at follow-up. Table S1. T cell responses by KS stage and gender at baseline.

## References

[1] Cevik M., Kuppalli K., Kindrachuk J., Peiris M. (2020). Virology, transmission, and pathogenesis of SARS-CoV-2. *BMJ*.

[2] Singh S., McNab C., Olson R. M. (2021). How an outbreak became a pandemic: a chronological analysis of crucial junctures and international obligations in the early months of the COVID-19 pandemic. *The Lancet*.

[3] Chanda D., Minchella P. A., Kampamba D. (2021). COVID-19 severity and COVID-19-associated deaths among hospitalized patients with HIV infection - Zambia, March-December 2020. *MMWR. Morbidity and Mortality Weekly Report*.

[4] Nasrullah A., Patel S., Ud Din M. T. (2021). A case of acquired immunodeficiency syndrome-related Kaposi sarcoma in a patient with COVID-19 - a brief review of HIV-COVID co-infection and its therapeutic challenges!. *Respiratory Medicine Case Reports*.

[5] Berenguer J., Diez C., Martin-Vicente M. (2021). Prevalence and factors associated with SARS-CoV-2 seropositivity in the Spanish HIV Research Network Cohort. *Clinical Microbiology and Infection*.

[6] Cele S., Karim F., Lustig G. (2021). *SARS-CoV-2 evolved during advanced HIV disease immunosuppression has Beta-like escape of vaccine and Delta infection elicited immunity*.

[7] Vidya Vijayan K. K., Karthigeyan K. P., Tripathi S. P., Hanna L. E. (2017). Pathophysiology of CD4+ T-cell depletion in HIV-1 and HIV-2 infections. *Frontiers in Immunology*.

[8] Ngalamika O., Munsaka S. (2020). Cells of the innate and adaptive immune systems in Kaposi's sarcoma. *Journal of Immunology Research*.

[9] Boasso A., Herbeuval J. P., Hardy A. W. (2007). HIV inhibits CD4+ T-cell proliferation by inducing indoleamine 2,3-dioxygenase in plasmacytoid dendritic cells. *Blood*.

[10] Cesarman E., Damania B., Krown S. E., Martin J., Bower M., Whitby D. (2019). Kaposi sarcoma. *Nature Reviews. Disease Primers*.

[11] Li Z., Wei Y., Zhu G., Wang M., Zhang L. (2022). Cancers and COVID-19 risk: a Mendelian randomization study. *Cancers*.

[12] Tsai H. F., Hsu P. N. (2017). Cancer immunotherapy by targeting immune checkpoints: mechanism of T cell dysfunction in cancer immunity and new therapeutic targets. *Journal of Biomedical Science*.

[13] Swain S. L., McKinstry K. K., Strutt T. M. (2012). Expanding roles for CD4(+) T cells in immunity to viruses. *Nature Reviews. Immunology*.

[14] Walls A. C., Park Y. J., Tortorici M. A., Wall A., McGuire A. T., Veesler D. (2020). Structure, function, and antigenicity of the SARS-CoV-2 spike glycoprotein. *Cell*.

[15] Cascarina S. M., Ross E. D. (2020). A proposed role for the SARS-CoV-2 nucleocapsid protein in the formation and regulation of biomolecular condensates. *The FASEB Journal*.

[16] Dutta N. K., Mazumdar K., Gordy J. T. (2020). The nucleocapsid protein of SARS-CoV-2: a target for vaccine development. *Journal of virology*.

[17] Boson B., Legros V., Zhou B. (2021). The SARS-CoV-2 envelope and membrane proteins modulate maturation and retention of the spike protein, allowing assembly of virus-like particles. *The Journal of Biological Chemistry*.

[18] Ngalamika O., Kawimbe M., Mukasine M. C. (2021). Expression of CD40L on CD4(+)T cells distinguishes active versus inactive HIV-associated Kaposi’s sarcoma. *Cancer Treat Res Commun*.

[19] Zitvogel L., Apetoh L., Ghiringhelli F., Kroemer G. (2008). Immunological aspects of cancer chemotherapy. *Nature Reviews. Immunology*.

[20] Sette A., Crotty S. (2021). Adaptive immunity to SARS-CoV-2 and COVID-19. *Cell*.

[21] Deeks S. G. (2011). HIV infection, inflammation, immunosenescence, and aging. *Annual Review of Medicine*.

[22] Martin M. A., VanInsberghe D., Koelle K. (2021). Insights from SARS-CoV-2 sequences. *Science*.

[23] Riou C., du Bruyn E., Stek C. (2021). Relationship of SARS-CoV-2-specific CD4 response to COVID-19 severity and impact of HIV-1 and tuberculosis coinfection. *The Journal of clinical investigation*.

[24] Alrubayyi A., Gea-Mallorqui E., Touizer E. (2021). Characterization of humoral and SARS-CoV-2 specific T cell responses in people living with HIV. *Nature Communications*.

[25] Biram A., Shulman Z. (2020). T cell help to B cells: cognate and atypical interactions in peripheral and intestinal lymphoid tissues. *Immunological Reviews*.

[26] Laidlaw B. J., Craft J. E., Kaech S. M. (2016). The multifaceted role of CD4(+) T cells in CD8(+) T cell memory. *Nature Reviews. Immunology*.

[27] Fendler A., Au L., Shepherd S. (2021). Functional antibody and T-cell immunity following SARS-CoV-2 infection, including by variants of concern, in patients with cancer: the CAPTURE study. *Research Square*.

[28] Marasco V., Carniti C., Guidetti A. (2022). T-cell immune response after mRNA SARS-CoV-2 vaccines is frequently detected also in the absence of seroconversion in patients with lymphoid malignancies. *British Journal of Haematology*.

[29] Tso F. Y., Lidenge S. J., Pena P. B. (2021). High prevalence of pre-existing serological cross-reactivity against severe acute respiratory syndrome coronavirus-2 (SARS-CoV-2) in sub-Saharan Africa. *International Journal of Infectious Diseases*.

[30] Mateus J., Grifoni A., Tarke A. (2020). Selective and cross-reactive SARS-CoV-2 T cell epitopes in unexposed humans. *Science*.

[31] Long Q. X., Tang X. J., Shi Q. L. (2020). Clinical and immunological assessment of asymptomatic SARS-CoV-2 infections. *Nature Medicine*.

