# Supplementary material for: SARS-CoV-2-Specific T Cell Immunity in HIV-Associated Kaposi Sarcoma Patients in Zambia
Source: J Immunol Res. 2022 Jul 28;2022:2114285. doi: 10.1155/2022/2114285 (PMC9352483; doi:10.1155/2022/2114285)
Supplement: Supplementary Materials — Figure S1. T cell responses in individual wells showing the spot-forming units. (a) Responses in a well for one of the peptide pools under investigation; (b) a negative control well where DMSO was added instead of peptide pools; (c) a well showing responses in the positive control well where stimulation of CD3, a T cell co-receptor, was done. Figure S2. Gating strategies used for immunophenotyping by flow cytometry. (a) Forward and side scatter with a gate on lymphocytes; (b) gating on CD3+ cells; (c) gating of CD4+ and CD8+ cells among the CD3+ cells; (d) subsets among the CD4 or CD8 cells including effector memory (Q1), central memory (Q2), naïve (Q3), and effector (Q4) cells. At least 100,000 events were collected in the lymphocyte gate. Figure S3. Correlation between CD4 counts with SARS-CoV-2-specific T cell responses. (a) a significant positive correlation between CD4 counts and T cell responses to spike at baseline; (b) a significant positive correlation between CD4 counts and T cell responses to NMO at baseline; (c) a significant positive correlation between CD4 counts and T cell responses to spike at follow-up; (d) a significant positive correlation between CD4 counts and T cell responses to NMO at follow-up. Table S2. Correlation between chemotherapy doses received and responses to SARS-CoV-2 at follow-up. Table S1. T cell responses by KS stage and gender at baseline. [file 2114285.f1.docx]

**Title:** SARS-CoV-2-Specific T-Cell Immunity in HIV-associated Kaposi Sarcoma Patients in Zambia


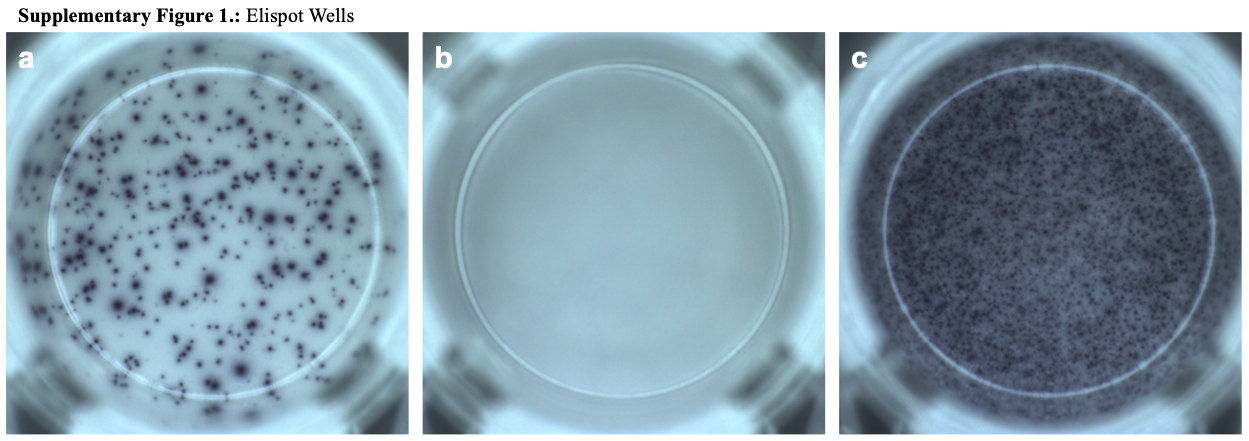


**Figure S1**. T cell responses in individual wells showing the spot-forming units. **a**) Responses in a well for one of the peptide pools under investigation; **b**) a negative control well where DMSO was added instead of peptide pools; **c**) a well showing responses in the positive control well where stimulation of CD3, a T cell co-receptor, was done.


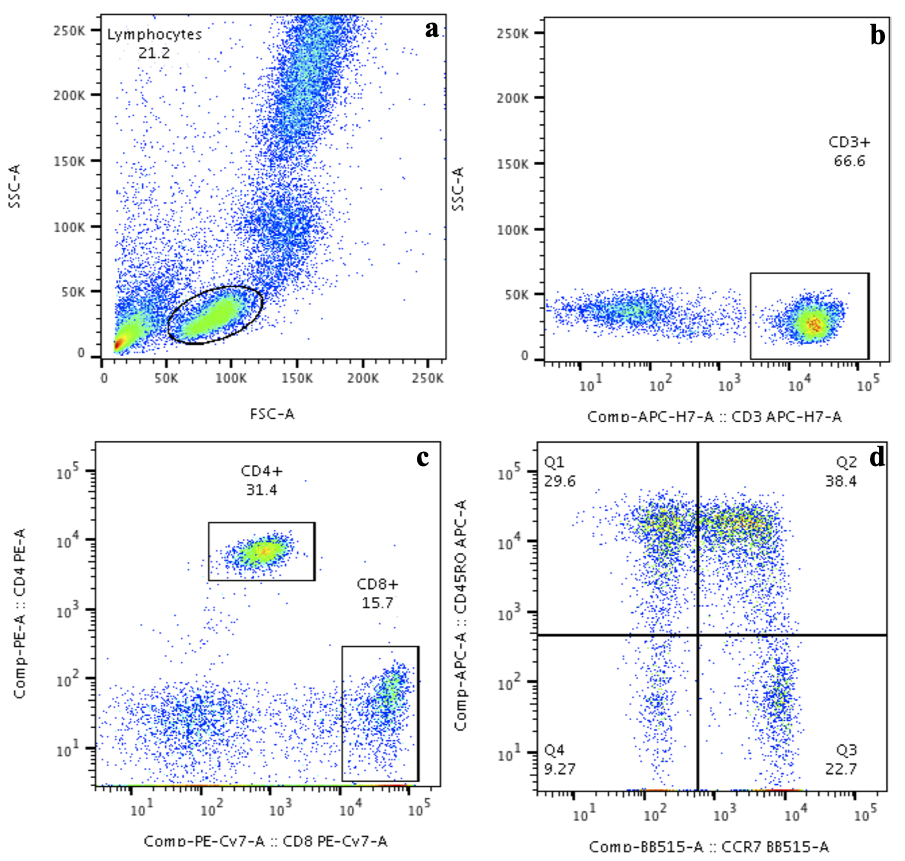


**Figure S2**. Gating strategies used for immunophenotyping by flow cytometry. **a**) Forward and side scatter with a gate on lymphocytes; **b**) gating on CD3^+^ cells; **c**) gating of CD4^+^ and CD8^+^ cells among the CD3+ cells; **d**) subsets among the CD4 or CD8 cells including effector memory (Q1), central memory (Q2), naïve (Q3), and effector (Q4) cells. At least 100,000 events were collected in the lymphocyte gate.


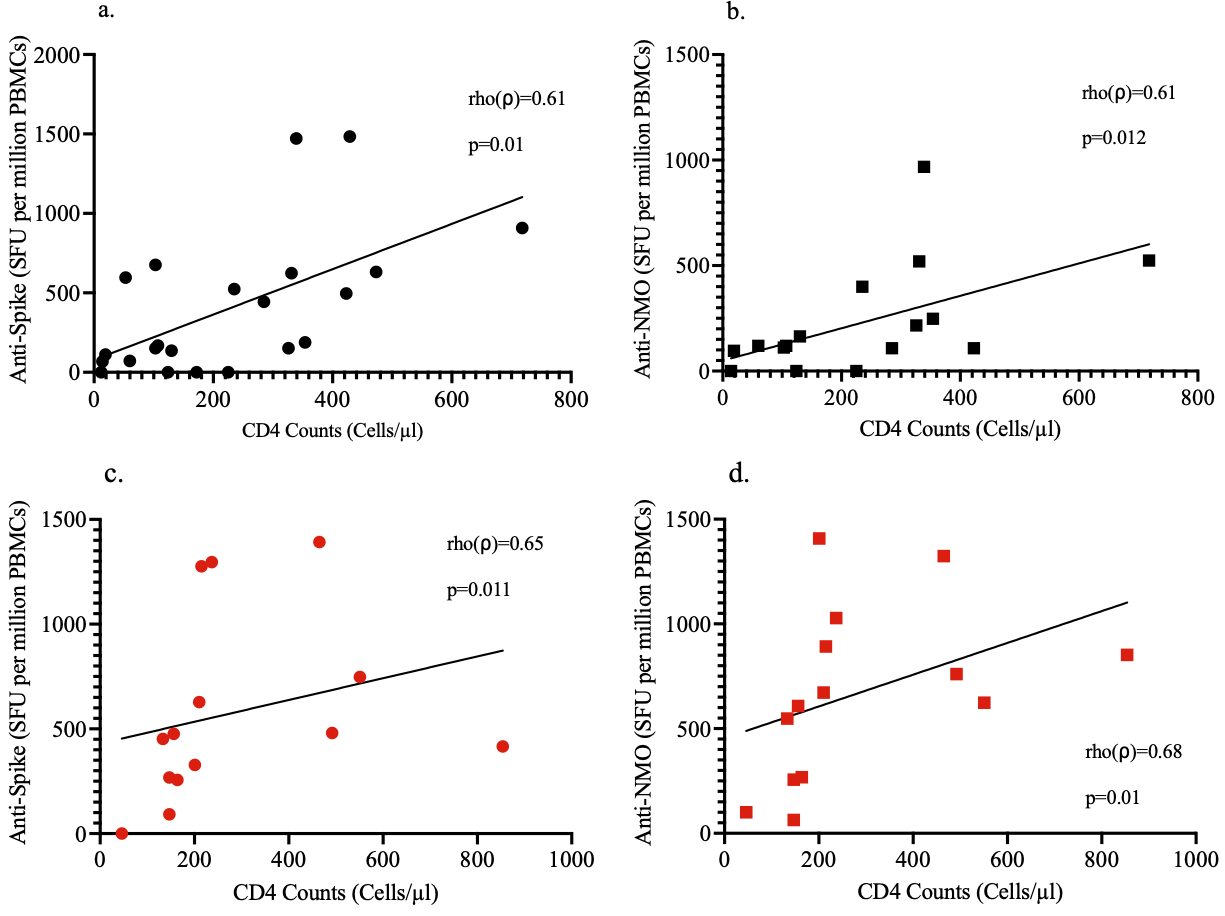


| **Table S1**. T Cell Responses by KS Stage and Gender at Baseline | | | | | | |
| --- | --- | --- | --- | --- | --- | --- |
|  | **KS Stage** | | | **Gender** | | |
|  | **T0 Stage**  **(N=9)** | **T1 Stage**  **(N=13)** | p value | **Male**  **(N=17)** | **Female**  **(N=5)** | p value |
| **Spike S1 Subunit** | 152SFU[72-444] | 496SFU[112-676] | 0.27 | 188SFU[68-596] | 168SFU[152-632] | 0.51 |
| **NMO** | 120SFU[108-248] | 120SFU[96-520] | 0.90 | 142SFU[54-324] | 116SFU[104-544] | 0.89 |
| T0 and T1 refer to the ACTG staging criteria; SFU=Spot-forming Units per million PBMCs; NMO=Nucleocapsid, Membrane protein, Open Reading Frame | | | | | | |

**Figure S3**. Correlation between CD4 counts with SARS-CoV-2-specific T cell responses. **a**) a significant positive correlation between CD4 counts and T cell responses to spike at baseline; **b**) a significant positive correlation between CD4 counts and T cell responses to NMO at baseline; **c**) a significant positive correlation between CD4 counts and T cell responses to spike at follow-up; **d**) a significant positive correlation between CD4 counts and T cell responses to NMO at follow-up.

| **Table S2**. Correlation between Chemotherapy doses received and Responses to SARS-CoV-2 at Follow-up | | |
| --- | --- | --- |
|  | Rho(⍴) | p value |
| **Spike S1 Subunit** | 0.31 | 0.28 |
| **NMO** | 0.10 | 0.74 |
| ⍴: Spearman’s Correlation Coefficient. | | |
